# The red ear syndrome

**DOI:** 10.1186/1129-2377-14-83

**Published:** 2013-10-04

**Authors:** Giorgio Lambru, Sarah Miller, Manjit S Matharu

**Affiliations:** 1 Institute of Neurology and The National Hospital for Neurology and Neurosurgery, Queen Square, London WC1N 3BG, UK

**Keywords:** Red ear syndrome, Migraine, Trigemino-autonomic reflex, Trigeminal autonomic cephalalgias, Parasympathetic system, Erythromelalgia

## Abstract

Red Ear Syndrome (RES) is a very rare disorder, with approximately 100 published cases in the medical literature. Red ear (RE) episodes are characterised by unilateral or bilateral attacks of paroxysmal burning sensations and reddening of the external ear. The duration of these episodes ranges from a few seconds to several hours. The attacks occur with a frequency ranging from several a day to a few per year. Episodes can occur spontaneously or be triggered, most frequently by rubbing or touching the ear, heat or cold, chewing, brushing of the hair, neck movements or exertion. Early-onset idiopathic RES seems to be associated with migraine, whereas late-onset idiopathic forms have been reported in association with trigeminal autonomic cephalalgias (TACs). Secondary forms of RES occur with upper cervical spine disorders or temporo-mandibular joint dysfunction. RES is regarded refractory to medical treatments, although some migraine preventative treatments have shown moderate benefit mainly in patients with migraine-related attacks. The pathophysiology of RES is still unclear but several hypotheses involving peripheral or central nervous system mechanisms have been proposed.

## Review

### Introduction

Red Ear Syndrome (RES) is a rare condition originally described by Lance in 1994 [1] and further characterized in 1996 [2]. Since the initial description, approximately 100 cases have been described in the literature [3-21]. The syndrome itself has not been clearly defined. Apart from its unique clinical presentation, characterised by episodes of burning pain and reddening of the external ear, the condition lacks definition in terms of aetiology, pathophysiology and treatments. It is for these reasons that it has yet to be included in the revised International Classification of Headache Disorders (ICHD-IIIβ) [22]. This article reviews the case descriptions, pathophysiological theories and effective treatments reported in the literature since the description of the first case in 1994.

### Epidemiology

The prevalence and incidence of RES is unknown, although the low number of reported cases would suggest that it is rare. There are 100 relatively complete case descriptions of RES found in the English language literature. The disorder has a slight female preponderance (54 women, 47 men), with a male to female ratio of 1:1.25. Interestingly, though, in the two series of young migraineurs with RES described by Raieli *et al.*[5,13], the majority of patients were males (75% and 68% of patients respectively). From the available data (ages of onset not provided by Raieli *et al.*[13]), the median age of onset of RES is 44 years old, although a wide range of 4 to 92 years is reported.

### Clinical features

#### Site of pain

Pain is felt over the pinna of the ear, often maximal on the ear lobe. It can radiate towards the cheek and mandible [16,20], as well as towards the occiput [4] and the involvement of the V1 area or whole hemicranium has also been described [1,10,12].

#### Laterality of attack

From the original description, RE episodes were thought to be predominantly unilateral and side-locked [1]. Subsequently, cases of unilateral attacks occurring on either side in the same patient [16] as well as bilateral attacks have been described [5,11,17]. In total, 62 patients (62%) had strictly unilateral attacks, 31 (31%) bilateral attacks and 6 (6%) either unilateral or bilateral attacks. Data is missing for one patient.

Among patients with unilateral RE episodes, the pain is reported more frequently on the left (58%) than the right side (42%) (left-sided attacks: 30 patients; right-sided attacks: 22 patients; data not available for 10 patients).

#### Severity of pain

The degree of intensity of the pain episodes in RES has not been properly assessed. Generally, it is reported as an annoying discomfort rather than an excruciating pain. However, authors do also report cases of patients complaining of moderate to severe attacks. Lambru *et al.* described a case of a patient with SUNA (Short-lasting unilateral neuralgiform headache attacks with autonomic symptoms) and RES occurring on the same side. The severity of the RE episodes were felt by the patient to be moderate (VRS: 5/10) compared to the excruciating intensity of the SUNA attacks (VRS: 8-10/10), suggesting a lower degree of severity of the pain of an RE episode compared to that typical of a TAC attack [23]. However, some authors have reported cases of patients complaining of severe pain. Boes *et al.* described a patient with episodes of left ear pain attacks associated with reddening that the patient described as severe. The attacks were suppressed completely with indometacin 75 mg/day, supporting a diagnosis of paroxysmal hemicrania-associated RES [3].

#### Character of pain

Patients consistently described the pain as a burning sensation. Other characters that have been less often reported include a dull ache [1,4], a stabbing pain [2,3], a sharp pain [2,10] and a jabbing pain [2].

#### Duration of the individual attacks

The duration of each RE episode can vary widely. Generally, the attacks are short-lasting with 63 RES patients (63%) reporting attacks lasting up to four hours. The majority had attacks lasting 30 to 60 minutes [2,5,16]. However, short-lived attacks lasting seconds have been reported [7]. Only six patients (6%) had attacks lasting over 4 hours of whom two complained of constant pain [2]. Data on duration of attacks in 32 patients was not available from the case reported.

#### Attack frequency and remission periods

The attack frequency varies immensely both among sufferers and within individual sufferers. The majority of RES patients report daily attacks ranging from one daily to up to 20 attacks a day [9]. A small minority of patients only report infrequent attacks that occur 1 to 6 times per month [5].

RE episodes occur most commonly during the daytime, although Lance reported a case in whom RE attacks had awakened the patient from sleep [2].

In most patients, RES seems to be a chronic problem without significant remission periods, although, in some cases the follow-up was too short to allow any meaningful conclusion to be drawn [7,15]. Some case reports clearly describe patients with episodic bouts of RES attacks alternating with remission periods. Donnet and Valade described a 92-year-old woman who experienced attacks of burning pain and red ear associated with lacrimation following an episodic pattern. The episodes were short-lasting, occurring in bouts of daily attacks lasting 15 to 45 days every 12–18 months [6]. Similarly, the case of a 36-year-old woman who complained of episodes of ear, temple, cheek and upper neck pain associated with ear redness and other autonomic symptoms such as conjunctival injection, tearing and nasal blockage lasting between 10–60 minutes was described. She had daily attacks occurring in “clusters”, presumably alternating with remission periods [16]. Finally Boulton *et al.* described the case of a 66 year-old woman with a five year history of episodes of pain behind the left ear and ear redness, initially occurring in bouts of daily attacks lasting 1–2 weeks, followed by remissions lasing months but that subsequently became almost daily [10].

#### Associated features

The key clinical feature of RES is the presence of burning ear pain associated with marked ear reddening although Hirsch reported five patients with episodes of bright red discoloration of the ears not associated with auricular pain [18]. The erythema of the ear consistently follows the ear pain and usually lasts as long as the burning pain does. Rare exceptions to this pattern exist. Selekler *et al.* described the case of a patient in whom the discoloration of the ear would disappear within 10 min from the onset of the attack, whereas the burning pain would continue for a further 5–10 minutes [11] and Boulton *et al.* reported a case of a 66 year-old woman in whom episodes of pain behind the left ear were accompanied by reddening of the ipsilateral ear only 50% of the time [10].

The area of reddening is usually limited to the external ear and is most pronounced over the earlobe [2,5]. Similarly to the pain pattern, the redness can extend beyond the ear, most frequently involving the cheek [2,16]. Involvements of the temple, upper neck and rarely the entire face ipsilaterally to the affected ear have also been reported [2,12,16]. Given the complex sensory and autonomic innervation of the external ear, it would be important to obtain more precise information on the area of reddening to help clarify the pathophysiological mechanisms at play. Unfortunately, besides some descriptions of redness involving only the earlobe and one case in whom the redness involved the helices [17], very little anatomical detail has been provided in the literature.

In the vast majority of RES patients, redness and warmth of the ear are the only associated symptoms. However, swelling of the ear during attacks has been reported in three cases [2,17,19] and other cranial autonomic symptoms such as aural fullness [3,9], ipsilateral lacrimation [21] and ipsilateral conjunctival injection, lacrimation and nasal blockage have also featured in the literature [2,3,6,16]. Nausea, vomiting, photophobia, phonophobia, osmophobia, motion sensitivity or restlessness are not features of RES.

#### Triggers

**Figure 1:**
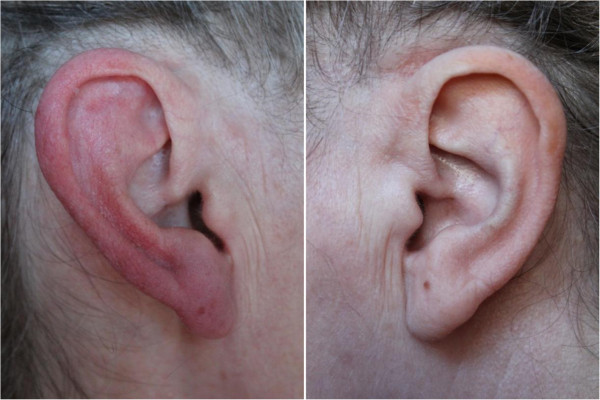
A red ear syndrome attack, provoked by rubbing her right ear.

**Figure 2:**
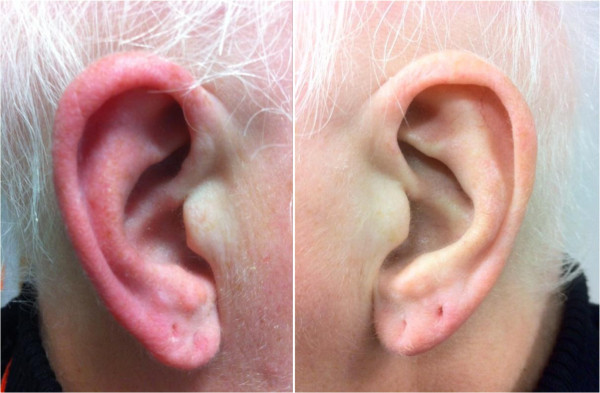
A spontaneous red ear syndrome attack, in a patient with migraine.

Most patients with RES have both spontaneous and triggered attacks. However, some patients can exhibit exclusively spontaneous attacks (25 patients) [5,9,14] or triggered attacks (14 patients) [2,7,11]. Triggers most often include: heat (reported in six patients) [2,10,15,19,20], rubbing the ear (three patients) [7,15,23], physical exercise (three patients) [2,9,20] and neck movement (three patients) [2,7]. Other cutaneous stimulations able to provoke RE episodes include: light touching of the ear, brushing of the hair, chewing, tooth grinding and showering [2,10,16]. Figure 1 shows a RE attack triggered by gentle rubbing of the ear and Figure 2 a spontaneous attack in a different patient.

### Anatomy, physiology and pathophysiology

Any attempt to define the pathophysiology of RES, must explain the burning ear pain along with ear flushing as well as the triggers of tactile stimuli and warmth. For this, an appreciation of the neural and vascular supply of the ear is essential.

The earlobe and the lower external aspect of the ear are innervated by the greater auricular nerve, a superficial branch of the C2-C3 spinal nerves, whereas the tragus and the anterosuperior aspect of the ear are supplied by the auriculotemporal branch of the mandibular division of the trigeminal nerve. The vascular supply of the ear is provided by branches of the middle temporal and posterior auricular arteries, which, as part of the external carotid circulation has sensory innervation of the blood supply from the trigeminal nerve. The dominant mechanism of vasomotor control of the skin of the ear is from variations in sympathetic vasoconstrictor tone, whereas the facial parasympathetic system seems to be only a marginal component accounting mainly for forehead and cheek skin vasodilation [24]. Importantly, vasodilation of the skin of the ear seems to be provoked by an inhibition of the sympathetic vasoconstrictor fibres as opposed to activation of the facial parasympathetic fibres.

The pathophysiological mechanisms underlying RES are currently unclear. However, several theories have been proposed and can be divided into two main groups namely, *peripheral theories* with mechanisms involving a dysfunction in cervical spinal nerves (predominantly C3 root) and *central theories* in which the underlying mechanisms would involve a dysregulation of brainstem trigemino-autonomic circuits.

In his original paper, Lance observed that RES was commonly associated with irritative lesions of the third cervical nerve root and this led him to suggest that, in cases of underlying cervical pathology, an antidromic discharge of impulses along C3 may occur leading to pain and vasodilatation due to release of vasodilator peptides [2]. Support for this hypothesis appeared to come from the fact that symptoms were temporarily relieved in one patient following local anaesthetic block of the C3 root and permanently relieved in another patient after C3 section. Lance also presented two cases in which attacks were triggered but where the underlying cause was thought to be TMJ dysfunction. In these cases he proposed that a local axon reflex, triggered by non-noxious stimuli such as heat, touch or chewing, might precipitate the antidromic discharge. He postulated that RES might be an example of Angry Back-firing C-nociceptors syndrome, in which the phenomenon of ‘cross-modality threshold stimulation’ occurs, whereby temperature changes alter the threshold for the pain induced by mechanical stimulation [25]. It was this hypothesis of an antidromic discharge from fibres of the third cervical root causing vasodilation in the referred pain area by discharge of vasodilator peptides, which led Lance to label RES an auriculo-autonomic cephalgia, though the exact mechanism by which C-fibres become “angry” was not specified [26].

On the basis of the presence of RES features in a patient with paroxysmal hemicrania, Goadsby and Lipton suggested that both conditions may share common pathogenic mechanisms centred on the brainstem connections between the trigeminal nerve and the facial parasympathetic outflow. They suggested a primary role for the dysregulation of brainstem trigemino-autonomic circuits in the pathophysiology of the syndrome [27]. The anatomical basis of this unifying hypothesis would be the convergence of processing nociceptive information at the level of the trigeminal nucleus caudalis and dorsal horn nuclei of the upper cervical spine, of both trigeminal afferents and C1-C2 spinal afferents. RES has subsequently been described in association with CH [16] and SUNA [23], thereby supporting the possible nosological and pathophysiological link between RES and TACs.

Given the frequent association of RES with migraine, Raieli *et al.* proposed that symptoms of migraine-associated RES may be due to the trigeminovascular activation during migraine attacks producing extracerebral vasodilatation via direct release of vasodilator substances (substance P, CGRP and nitric oxide) [5]. The activation of the trigeminovascular system would explain the ear pain that extends beyond the trigeminal distribution to the earlobe, due to the overlap between trigeminal and upper cervical spinal nerves in the trigemino-cervical complex [28]. The same authors showed that RES was associated with migraine features partially provoked by parasympathetic system activation and concluded that RES could be considered a specific sign of parasympathetic hyperactivation, via the trigemino-autonomic reflex, during migraine, in essence considering RES as a migraine-related phenomenon, sharing activation of pathophysiological mechanisms known to be pivotal in migraine.

However, all these theories concerning a trigemino-autonomic dysregulation as the main mechanism in RES and RES associated with migraine and TACs raise an interesting anatomical inconsistency. The trigemino-autonomic reflex is based on a brainstem connection between the trigeminal nerve and the facial parasympathetic outflow [27]. According to this hypothesis, the facial nerve parasympathetic outflow fibres that appear to be responsible for the facial autonomic symptomatology in TACs should also be able to produce vasodilation and therefore redness of the skin of the ear in RES. However, unlike other parts of the face, such as the cheek and the nose, the vasodilation of the skin of the ear is mainly under sympathetic vasoconstrictor control and occurs when there is inhibition of the sympathetic vasoconstrictor fibres [24]. During TACs attacks there is marked trigemino-parasympathetic activation, along with a sympathetic deficit. It may be possible that in cases of RES in conjunction with TACs, the presence of an imbalance between parasympathetic and sympathetic systems in the latter may in turn facilitate the inhibition of the sympathetic tone of the ear giving raise to the red ear phenomenon. However, as it is the sympathetic dysregulation and not a parasympathetic activation that is the predominant mechanism for the ear reddening, it seems less likely that the trigemino-autonomic reflex plays a central role at least in isolated cases of RES. Indeed, other mechanisms may contribute more specifically to the red ear phenomenon.

A further pathophysiological hypothesis is based on the fact that the clinical presentation of RES is strikingly similar to the clinical presentation of erythromelalgia (EM), a dermatological condition characterised by paroxysmal episodes of burning pain and erythema often involving hands and feet. The diagnostic criteria of EM include: burning pain, aggravated by warmth and relieved by coldness, erythema and increased skin temperature [29]. The pathophysiology of EM is unfortunately unclear but local primary damage of vascular and neuronal structures has been proposed as the main pathophysiological mechanism underlying it. In essence, it is postulated that primary vascular misdistribution leading to skin hypoxia may cause a secondary hypoxic-induced neuropathy. Conversely, primary small-fibre dysfunction may lead to a secondary vascular misdistribution and hypoxia accompanied by secondary capillary proliferation [30,31]. Given the clinical similarities, some authors have raised the possibility that RES may be an auricular variant of EM, possibly caused by similar small sensory and sympathetic nerve dysfunction [19]. Moreover, they stated that since a gene has been identified in hereditary EM caused by mutations of Na 1.7 channels in sensory and sympathetic nerves [32], sodium-channel blocking drugs may be a reasonable therapeutic option to explore in the management of RES.

### Secondary RES

The majority of RES described in the medical literature are primary but secondary RES has been reported (25 cases). Lance originally described the first series of 12 patients with RES. Ten out of these patients had a secondary pathology thought to be responsible for the RE episodes [2]. Subsequently other authors have reported cases of RES secondary to an underlying pathology (Table 1) [7,9,10,16].

**Table 1:** Secondary causes of red ear syndrome

**Upper cervical spine**	• Hypertrophy of the ipsilateral C2-C3 facet joint
	• Degeneration of superior facet of C4
	• Cervical arachnoiditis with posterior column myelomalacia
	• Traction injury of upper cervical roots
	• Narrowing of C4 neural foramen
	• Chari I malformation
	• Chronic whiplash
	• Congenital fusion of C1-C3 vertebrae with enlargement of the cervical spinal canal
	• Neurovascular compression between vertebral artery and C3 root
**Cranial and cervical neuralgias**	• Atypical glossopharyngeal neuralgia
	• Atypical trigeminal neuralgia
	• C3 root neuralgia
**TMJ dysfunction**	
**Thalamic syndrome**	
**Herpes zoster**	
**Pleomorphic adenoma of carotid body**	

Secondary RES cases can be divided in two main groups: upper cervical spine lesions and temporo-mandibular joint dysfunction (TMJD). Eleven patients with RES secondary to upper cervical spine abnormalities were found in the literature. Different aetiologies have been implicated including cervical spondylosis, infections of the meninges, traction injury, narrowing of the neural foramen at the side of the pain and congenital cervical abnormalities (Table 1). Eleven patients with RES and co-existing TMJD ipsilateral to the side of the pain have been described [2,9,10,16]. One patient’s RES attacks settled with the use of a dental plate seeming to point to a causal relationship between TMJD and RES in this case [2].

Other secondary causes of RES include Chiari 1 malformation, thalamic syndrome and a neurovascular compression of the C3 root by the vertebral artery [2,9,21].

### Investigations

Due to the association of secondary RES with upper cervical spine pathology and TMJD, an MRI of the cervical spine should be carried out and, when the clinical suspicion for TMJD is high, an orthodontic assessment is warranted. In addition, given the possible link between RES and thalamic lesions [2,17], an MRI scan of the brain should be included in the diagnostic work up of RES.

Given the description of a patient with chronic paroxysmal hemicrania (CPH), presenting as RES with unilateral short-lasting, attacks of ear pain and redness, sometimes involving the entire side of the face which responded to indometacin [3], all patients with short-lasting strictly unilateral side-locked attacks of ear pain and redness occurring more than once a day should have a trial of indometacin up to 225 mg a day [33], to exclude an indometacin-sensitive headache disorder.

### Treatment

Several of the drugs routinely used in primary headaches and other pain syndromes have been tried in cases or small series of RES patients in an open-label fashion. Most of these drugs have been reported produce a marginal benefit only. RES is, therefore, generally considered refractory to treatments. Medications with some reported effect are summarised in Table 2.

**Table 2:** Effective treatments in red ear syndrome

	**Number of patients**	**Type of RES**	**Dosage**	**Improvement**
**Gabapentin**	7	Primary and secondary	-	Good
**Ice pack**	4	Primary	-	Good
**Amitriptyline**	3	Primary and secondary	-	Good
**Indometacin**	2	Primary	50-75 mg/day	1 patient: pain free
				1 patient: good
**Imipramine**	1	Secondary	125 mg/day	50% reduction in pain severity
**Verapamil**	1	Secondary	-	-
**Ibuprofen**	1	Primary	400 mg	Pain free
**Propranolol**	1	Secondary	80 mg	Good
**Flunarizine**	1	Primary	-	-
**Nimodipine**	1	Primary	-	-
**Pregabalin**	1	Primary	600 mg	Mild
**Methysergide**	1	Secondary	-	-
**Greater auricular nerve blockade**	1	Primary	2% prilocaine 1 cc + 125 mg methylprednisolone	Pain free

#### Non-steroidal anti-inflammatory drugs

Ibuprofen taken daily was reported to be highly effective in two patients with RES [10]. In one of these cases, the benefits persisted even when the drug was discontinued due to side effects, raising the possibility of a spontaneous improvement of the condition rather than a specific effect of the medication. Conversely, ibuprofen 1200 mg for 7 days has had no effect in other cases [15]. Oral indometacin given at the dose of 25 mg tds, completely suppressed the pain in the patient with CPH-related RES [3] but when tried in a dose up to 50 mg tds in other five patients with RES, only one reported an improvement [10,16].

#### Tricyclic antidepressants

Three patients were said to respond to a trial of amitriptyline but no details of dosing were given [9]. Imipramine 125 mg daily was effective in one patient, reducing pain severity by 50%, but no mention of any effect on ear redness was made [2].

#### Beta-blockers

The β-blocker propranolol was used at a dose of 40 mg twice daily to successfully treatment one case of RES secondary to cervical spondylosis. However, in another case of secondary RES associated with possible TMJD, propranolol was not found to be helpful [2].

#### Calcium channel blockers

Verapamil was reported to be effective in two RES patients. In both these cases, the syndrome was associated with other conditions such as chronic whiplash and TMJD. In one patient, verapamil was administered in combination with other drugs [16]. Flunarizine and nimodipine have also been tried in two patients with migraine-related RES. In both the cases, the treatments led to a reduction in frequency and severity of both the RE and migraine episodes [5].

#### Anticonvulsants

Gabapentin has been the most widely tried medication in RES patients to date. In a series of 12 patients with RES, seven of the eight on gabapentin reported a marked improvement in terms of frequency of attacks and ear colour changes. One patient with possible cluster headache-related RES also responded to a combination of gabapentin and verapamil although no information about dosages was given [16]. One patient failing gabapentin did obtain a slight reduction of pain using pregabalin 600 mg daily [12].

#### Serotonergic agonists and antagonists

Triptans have been tested in two patients with limited benefit reported [16]. One patient with RES secondary to cervical arachnoiditis, failed to respond to pizotifen, but did show improvement with methysergide [2].

#### Topical lidocaine or steroidal application

Topical applications of anaesthetics and steroids are generally ineffective. Two patients failed topical application of lidocaine [12,20] and two patients topical steroids (clobetasol 0.05% cream being used in one such case) [15,20]. However, cooling of the ear by application of an ice-pack has been reported to provide significant relief in five patients during an acute attack [2,5,15,19].

#### Local nerve blockade

Blocking the greater auricular nerve (GAN) with 1 cc prilocaine 2% and 125 mg methylprednisolone in a patient with RES led to a symptom free period of more than eight weeks. In this case, RE episodes were brought on by light touching of the ear and during these attacks the patient reported GAN tenderness [11]. Lance reported a poor outcome of C3 root blockades in two patients with RES. The block was performed in one patient using bupivacaine 0.5%, with pain relief lasting only few hours, whereas a C2 and C3 root block in the second patient relieved the pain for several hours [2].

### Classification and diagnostic criteria

Given the relatively limited description of RES in terms of aetiology, pathophysiology and treatment, it is currently not included in the ICHD-II [34] and ICHD-IIIβ [22]. Some authors have suggested that RES could be considered a form of TAC on the basis that both are a similar phenotype characterized by short-lasting attacks of unilateral pain, occurring once or more times a day associated with cranial autonomic features [27]. Lance proposed that since symptoms in RES are mostly centred over the earlobe (which derives its sensory supply from C3), RES may be a form of autonomic cephalalgia mediated by the greater auricular nerve rather than the trigeminal nerve, and that therefore, the term “auriculo-autonomic cephalgia” rather than TAC should be used [26].

RES often appears to occur in associated with migraine without aura. Indeed, 81% (n=60/74) of patients reported in the literature with primary RES had a personal history of migraine [5,6,9,13-16]. Given this striking association, Raieli *et al.*, suggested that RES be encompassed into the wider clinical spectrum of migraine, with RES episodes considered a migraine related phenomenon when occurring during a migraine attack and an “acephalgic” migraine phenomenon when occurring outside a migraine attack [5]. Subsequently, the same authors studied a group of 40 young migraineurs with RES and found that RES was significantly associated with some of the migraine associated features, possibly explained by parasympathetic nervous system activation. They concluded that RES could be considered a specific sign of parasympathetic hyperactivation via the trigemino-autonomic reflex, occurring during migraine [13].

In view of its various possible aetiologies, other authors argued that primary and secondary forms of RES be kept separated. They proposed that primary RES be considered a migraine-related phenomenon, whereas secondary forms considered a neuralgiform radiculopathy involving the C3 root [7].

Although the pathophysiological mechanisms and consequently the treatment of RES remains unclear, the clinical description in 100 patients provides enough detail to allow us to propose the first set of diagnostic criteria (Table 3). We propose that RES be included in the 4^th^ Chapter of the IHS Classification, “Other Primary Headache Disorders”, until further understanding regards its pathophysiology and treatment emerges. Since secondary forms of RES are rather frequent, physicians should carefully look for secondary causes of RES, namely a disease of the upper cervical spine and TMJ dysfunction. Secondary RES due to a disorder of the neck or TMJ should be classified within Chapter 11 of the ICHD-IIIβ (Headache or facial pain attributed to disorder of the cranium, neck, eyes, ears, nose, sinuses, teeth, mouth or other facial or cervical structure).

**Table 3:** Proposed diagnostic criteria for primary red ear syndrome

**A.**	At least 20 attacks fulfilling criteria B-E
**B.**	Episodes of external ear pain lasting up to 4 hours.
**C.**	The ear pain has at least two of the following characteristics:
	-Burning quality
	-Unilateral location
	-Mild to moderate severity
	-Triggered by cutaneous or thermal stimulation of the ear.
**D.**	The ear pain is accompanied by ipsilateral redness of the external ear.
**E.**	Attacks occur with a frequency of ≥1 per day, although cases with lower frequency may occur.
**F.**	Not attributed to another disorder.

### Primary versus secondary RES

Although primary and secondary forms of RES share common epidemiological and clinical characteristics, differences in age of onset, clinical presentation and associated conditions have been described. For this reasons, Donnet and Valade proposed to distinguish RES into two separate types - a primary form that occurs in younger people with a personal history of migraine, and a secondary form seen more frequently in older patients with upper cervical spine pathology or TACs [6]. The analysis of the published cases (Table 4) indicates that patients with primary RES have a younger age of onset compared to those with secondary RES and that whilst both forms show a female preponderance this is more pronounced in secondary RES. Clinically, a higher proportion of patients with the secondary form of RES have daily and triggerable attacks as compared to those with the primary form, a difference that may be attributable to the underlying pathology. As suggested by Donnet and Valade, a higher proportion of patients with primary RES had a personal history of migraine.

**Table 4:** Characteristics of primary and secondary forms of red ear syndrome (RES)

	**Primary RES**	**Secondary RES**
**Number**	74	26
Median age of onset^*^	34 years (range: 5–74)	45 years (range: 9–76)
**Gender**	Females: 37 (51%)	Females: 17 (65%)
	Males: 36 (49%)	Males: 9 (35%)
RE episodes: frequency^***^	≥ 1 attack/day: 17 (53%)	≥ 1 attack/day: 17 (77%)
	< 1 attack/day: 15 (47%)	< 1 attack/day: 5 (23%)
RE episodes: duration^**^	<4 hours: 47 (94%)	<4 hours: 13 (68%)
	>4 hours: 3 (6%)	>4 hours: 6 (32%)
**Triggered attacks**	16/74 (22%)	18/26 (69%)
**Personal history of migraine**	60/74 (81%)	6/26 (23%)

*The median age of onset in primary RES is based on 34 patients for whom clear cut data were available.

^**^Clear cut duration of attacks was available for 50 patients with primary RES and 19 patients with secondary RES.

^***^Data on attack frequency were available for 32 and 22 patients with respectively primary and secondary RES.

In summary, the primary form of RES affects younger males and females almost equally and is characterised by short-lasting episodes occurring with a variable frequency. Attacks are usually spontaneous and most patients have a personal history of migraine. The secondary form of RES occurs in older people, with a female preponderance, usually without a personal history of migraine and is characterised by short-lasting, daily attacks, often provoked by specific triggers (Table 3). More clinical and therapeutic data is needed to ascertain if primary and secondary forms show different responses to medical treatments, as suggested by some authors [10].

## Conclusion

The features of RES have been reviewed in 100 patients. RES is a rare but probably under-recognised condition characterised by unilateral or bilateral episodes of burning pain and erythema over the external ear. The episodes are usually short-lasting and frequent. On the basis of the clinical features described in literature, the first set of diagnostic criteria for RES has been proposed here.

Two different forms of RES can be distinguished: primary RES occurring in young individuals, often migraineurs, and characterised mainly by spontaneous attacks, sometimes temporally related to a migraine attack; and, secondary RES occurring in older subjects with attacks more likely provoked by cutaneous triggers, heat or chewing. Symptomatic causes often responsible for secondary RES include upper cervical spine lesions and TMJD. The diagnostic workup including an MRI scan of the head and cervical spine, along with orthodontic assessment is recommended in these patients.

RES appears generally refractory to medical treatments. Open-label trials have suggested a beneficial effect with in some cases with the use of gabapentin, amitriptyline, imipramine, flunarizine, propranolol, verapamil and pregabalin. Other treatments including NSAIDs, ice-pack use and blockade of the greater auricular nerve can also be considered.

Peripheral and central theories of the pathophysiology of secondary and primary forms of RES have been proposed. More recently a primary dysfunction of small sensory and sympathetic fibres of the ear skin has been raised due to the clinical overlap with EM. This hypothesis might justify the use of medications that have not previously been tried in this condition, such as sodium channel blockers, and widen the therapeutic options for this syndrome.

## Consent

Written informed consent was obtained from the patients for the publication of this report and any accompanying images.

## Competing interests

There Authors declare that they have no competing interests. GL and SM have no disclosures. MSM serves on the advisory board for Allergan and St Jude Medical, and has received payment for the development of educational presentations from Allergan, Merck Sharpe and Dohme Ltd and Medtronic.

## Authors’ contributions

GL reviewed the literature and contributed in drafting the manuscript. SM contributed in drafting and revising the manuscript. MSM contributed in revising the manuscript. All authors read and approved the final manuscript.
